# Supplementary material for: Scientific prizes and the extraordinary growth of scientific topics
Source: Nat Commun. 2021 Oct 5;12:5619. doi: 10.1038/s41467-021-25712-2 (PMC8492701; doi:10.1038/s41467-021-25712-2)
Supplement: Supplementary file 1 — Supplementary Information [file 41467_2021_25712_MOESM1_ESM.pdf]

Scientific Prizes and the Extraordinary Growth of Scientific Topics

Authors: Ching Jin[1,2], Yifang Ma[1,3], Brian Uzzi*[1,2]

Affiliations:

[1]Northwestern Institute on Complex Systems (NICO), Northwestern University, Evanston IL, 60208, USA.
[2]Kellogg School of Management, Northwestern University, Evanston IL, 60208, USA.
[3]Department of Statistics and Data Science, Southern University of Science and Technology, Shenzhen, Guangdong 518055, China
*Corresponding Author: uzzi@northwestern.edu

Supplementary Information

1. Data Description:

Scientific Prizes and Scientific Topics. We collected a comprehensive dataset that combines 458 recognized scientific prizes with 12,041 scientific topics through prizewinning events occurred between 1970 and 2007. From these 12,041 prizewinning topics, more than 95% could be matched successfully with five non-prizewinning topics, results in our main dataset of 11,539 prizewinning topics and their related 405 prizes (for details see Sec. 4). The prizes in our sample include celebrated awards like the Wolf Prize and Turing Prize as well as hundreds of others recognized on Wikipedia's "scientific prizes" page. To validate the Wikipedia data, we manually cross-checked it with prize-related data on the web and in print media. To avoid misclassification of scientific prizes, a prize was considered a science prize if it had at least ten scientist-prizewinners following the Ma et al methodology.[1]

To link prizes awarded to scientists to scientific topics, we used Microsoft Academic Graph (MAG) data. MAG uses state of art NLP algorithms to classify 172,037,947 papers from 209,404,413 scientists to scientific topics before 2018[2,3]. The MAG associates papers with 228,251 topics (a.k.a "fields of study"), that are nested within 293 domains (e.g., Quantum Mechanics, Algebra, etc.), which are nested with 19 disciplines (e.g., Physics, Mathematics, etc.). Because the algorithm that assigns a paper to topics based on a paper's complete text and in relation to other texts, a paper's assigned topics are not synonymous with author-defined "keywords." For

example, the paper, “The human disease network[4],” has the keywords biological networks, complex networks, human genetics, systems biology, and diseasome and classified firstly in the topics: Human Ineractome and then network medicine.

The above process generated a list of research topics for each publication of every prizewinner. To avoid misclassification errors of topic-labeling in MAG and to make sure that the listed topics are a meaningful part of a scientist's reputation, we considered a topic to be associated with a scientist prizewinner only if the scientist published at least $L = 10$ papers on the topic. We validated this criterion using Wikipedia's “*known for”* dataset, which crowdsources scientist opinions about the topics that other scientists are known for. The *known for* data has 3,427 “known-for” topics for our prizewinners. The average number of publications of a scientist's known-for topics is 10.0252, corroborating $L=10$. After further matching with non-prizewinning topics, 405 prizes are identified to be linked to 11,539 prizewinning topics through 2,900 prize conferrals. This dataset has been used in our main analyses. We also use the full 458-prize dataset in different robustness tests when selecting $L = 5, 15,\ and\ 20$, finding consistent results (Supplementary Fig. S1).

NIH Grants: We collected NIH grant data from 1985 to 2015 that estimated the amount of funding a topic received before and after the prize year. Grants were linked to different research topics through the NIH publication list associated with each grant. By counting the number of NIH grant links, we obtained an estimated “NIH grant mention” for each topic. Because the total number of NIH grants change year-by-year ($G(t)$) and, by definition, the NIH mention will change with this number, we adjusted the NIH mention by normalizing it with the total number of NIH grants ($G(t)$)

$$Adjusted\ NIH\ mention\ (t) = \frac{NIH\ mention\ (t)}{G(t)} * < G(t) >$$

where $G(t)$ is the total number of NIH grants for year t, and $< G(t) >$ calculates the average total number of NIH grants for different years. We find the adjusted NIH mention for the prizewinning topic is flat before and after the prizewinning event, indicating the funding has no measurable influence on the post-prize growth.

2. Quantifying Growth Patterns of Research Topics

To study whether a growth pattern before and after a topic is associated with a topic's prizewinning event, we find five topics in the same discipline that had growth patterns that were statistically indistinguishable from the prizewinning topics for ten years before the prize year.

To quantify the comparative growth of the prizewinning relative to the peer topics, we defined Δ_t over time t:

$$\Delta_t = \log(Y_t) - \log(\widetilde{Y}_t).$$

which measures the differences in the logarithm of the quantities and provides an appropriate method for establishing percentages changes in a quantity (prizewinning topic's growth, Y_t) relative to a baseline (geometric mean of the peer topics growth, $\widetilde{Y}_t$) since quantities such as citations may have fat-tail distributions[5,6]. As it is well known, the absolute differences perform better for quantities with a narrow Gaussian like distribution, which can be achieved by taking the logarithm of the six quantities, leading to our logarithm difference measure.

Robustness Check: To make sure our results are not methodology-dependent, we also test our results with standard ratio difference measure[7,8] (Supplementary Fig.S2 a-f), which show growth patterns similar to the ones we present in the main text. A further placebo test also corroborates our main findings (Supplementary Fig.S2 g-l). Specifically, for each of the prizewinning topic, we selected one non-prizewinning topic from its peer topic candidate pool as a "pretend winning topic", repeating the analysis in Supplementary Fig.2 a-f, finding that there is no difference in the expected growth for pretend topic before and after the prizewinning event, supporting our main conclusions.

4. Dynamic Optimal Matching Method

To select the peer non-prizewinning topics, we use a Dynamic Optimal Matching method, which applies the Optimal Matching Method[9-11] to a time-series data to simultaneously maximize the

closeness and balance characteristics of accurate matching[10-13]. We focus on prizewinning between 1970 and 2007, resulting in a sample of 12,041 prizewinning and peer non-prizewinning topics. 11,539 topics (> 95%) could be statistically matched to peer topics using DOM. If a topic has multiple prizes over its lifetime, we match for the first prizewinning.

First, we select a peer topic candidate pool[9]. To achieve this, for each prizewinning topic i, we selected up to 40 close-distance topics in terms of a distance measure ($\theta_{i,j}$) from the same discipline, generating a peer candidate pool. For 95% of the prizewinning topics, a proper peer candidate pool was identified (11,539/12,041= 95.8%). To achieve matching, we defined a distance measure $\theta_{i,j}$ to quantify the closeness between the prizewinning topic i and a non-prizewinning topic j[7,8]:

$$\theta_{i,j} = \frac{\sum_{n=1}^{N} \sum_{t=t^*-t_0}^{t^*} \left(\log Y_{i,n}(t) - \log Y_{j,n}(t)\right)^2}{N * (t_0 + 1)}$$

, where $Y_{i,n}$ indicates the quantity for the topic i in terms of one of the N =6 matched categories (i.e. Productivity, Citations, Lead Scientist impact, #incumbents, #Entrants and #Disciplinary Stars). t measures number of years prior to the prizewinning year. t^* represents the prizewinning year for topic i, and $t_0 = 10$, indicates we traced the growth pattern for topics in an 11-year duration, which includes 10 years prior to the prizewinning year.

Second, to ensure the balance between the peer and prizewinning topics for the entire system, we select 5 matching topics from the candidate pool to be the topic's peer group. In this process, we (1) minimize the distances between the peer and prizewinning topics in terms of $\theta_{i,j}$, and (2) make sure the distribution of the peer and prizewinning topics are acceptably and simultaneously close for all 66 covariates.

Specifically, we make sure the differences between the prizewinning and peer topic groups are small enough for each matching category n, and for any time t before the prizewinning event ($-10 \leq t \leq 0$), where the differences between the prizewinning topic i and its expected growth at time t and category n are quantified by $\Delta_{i,n}(t) = \left(\log Y_{i,n}(t) - \log \tilde{Y}_{i,n}(t)\right)$. The expected growth is obtained by averaging the trajectory of the matched topics. This problem is a classical optimization problem, which could be solved with typical Mixed Integer Programming (MIP) methods[9,13]. We found the best-optimized matching possible where (1) the distance between the prizewinning topics and the peer topics is minimized; at the same

time, (2) the difference between the peer and prizewinning groups is not statistically significant for any $t \in [-10,0]$ and $n \in [1,6]$. Mathematically, we have:

$$\left|\frac{\sum_{i=1}^{M} \Delta_{i,n}(t)}{M} - 0\right| < 1.96 * SE(\Delta_{i,n}(t)).\ for\ \forall\ 1 \leq n \leq 6, -10 \leq t \leq 0$$

Here $SE(\Delta_{i,n}(t))$ measures the standard error of the $\Delta_{i,n}(t)$ for the prizewinning topics at time t and in category n, and M captures number of prizewinning topics. To prevent bias by topics with a large $\Delta_{i,n}(t)$ in the MIP process, we also monitored the topic-by-topic growth of each individual topic. Specifically, for any t $(-10 \leq t \leq 0)$, we compare the growth pattern of each prizewinning topic and all of its peer topics (11,539*5=57,695 pairs). For each of the 66 covariances, we ensure each prizewinning topic had equal probability to grow faster or slower than any of its peer topics. Supplementary Fig. S4 shows the binomial tests validating our method, demonstrating that all p-value is larger than 0.2. This method not only guarantees closeness between the peer and prizewinning topics but also unsure good balancing *between* and *within* topic groups.

To further validate our DOM method, we also run an additional "placebo" tests that pretend that each peer topic is a prizewinning topic with the purpose of testing whether the peer topics also showed abnormal growth following the prize year of the prizewinning topic (Supplementary Fig. S5). Specifically, for each of the prizewinning topic, we selected a non-prizewinning topic from its matching candidates as a "pretend winning topic", matched it with 5 peer topics, repeating the whole analysis in Fig. 2, finding that peer topics have no coincidental extraordinary growth (all *p*-values>0.05), reinforcing our main finding prizewinning is associated with and a topic's onset of a sustained period of extraordinary.

To account for possible correlations among different variables, we also repeat the analysis by adopting *Mahalanobis distance* to quantify the closeness of the topics. Specifically, we present the pre-prize growth patterns of a topic as a 66-element (6 categories * 11 years) vector $\vec{y_i}$, quantifying the logarithm growth of the topic i in the 11-years period before the prizewinning event in terms of the six measures. We can calculate the Mahalanobis distance between the prizewinning topic i and a non-prizewinning topic j:

$$\theta'_{i,j} = \sqrt{(\vec{y_i} - \vec{y_j})^T S^{-1} (\vec{y_i} - \vec{y_j})} \,,$$

where S is the covariance matrix. By adopting this distance measure in our matching procedure, we repeat the main analysis, finding consistent results (Supplementary Fig. S6).

5. Paradigmatic Diversification

Prizewinning topics become paradigmatically diverse than matched peer topics. Paradigmatic diversification refers to the heterogeneity of concepts scientists use to study a topic[14,15]. To measure paradigmatic diversification, we created a master list of all the topics new entrants in prizewinning or peer topics had published on before becoming a new entrant. Topics in the master list were defined as different from one another if topics were associated with different disciplines (N=19 disciplines). The topic diversity of the master list was then measured using Shannon Entropy as: $S = -\sum_j p_j \log_2 p_j$, where j represents a discipline, and p_j measures the probability that a topic in the list belongs to discipline j. We observe that paradigmatic diversity's distribution at $\Delta_{10} \geq 0$ for prizewinning topics is significantly more diverse than peer topics (K-S test, $\Delta_{10} \geq 0$ group, $p < 0.0001$). Supplementary Fig. S7 shows that when Δ_{10} equals zero (i.e., no growth differences), prizewinning and peer topics have no significant difference in paradigmatic diversification. However, as Δ_{10} grows, relative paradigmatic diversification increases significantly (slope $= 0.109,\ \ p < 0.0001$). For example, when Δ_{10} equals to 1.5, paradigmatic diversity is 11.6% greater for prizewinning topics than it is for peer topics.

6. Prize Characteristics Predict Magnitude of Extraordinary Growth

To examine the results' sensitivity to confounds, we regressed the Δ_{10} of our six growth variables on money, discipline-specific, and recency along with control variables. Control variables include lagged values of each growth trend at times *t*-1, *t*-2, and *t*-3 years to account for autoregressive effects of Δ_{10}. A disciplinary fixed effect variable controls for stable disciplinary differences such as prestige, theoretical vs bench science disciplines, and level of paradigm development. To control for yearly numbers of scientists and publications, we added a calendar year fixed effect. To account for differences in a prize's visibility, we added control variables for total Wikipedia pageviews of the prize (measured as of 2017), a binary variable for whether the

prizewinner is among the top 5% of cited authors on that topic, a binary variable for whether there are multiple prize recipients for the topic in the same year, number of prize conferrals up to the prize year, and the age of the prize up to the prize year. To better interpret the results, we show both results of the original variables (Supplementary Tab. S2) and the variables which have been standardized (Supplementary Tab. S3).

The results are further validated by adopting the *BIC* statistics, where we find signal strengths adding a large explanation to the extraordinary growth of prizewinning topics (Supplementary Tab. S4-S9). 10-fold cross-validation indicated that there was no overfitting of the regression model (Supplementary Tab. S10). Because the amount of prize money can range widely, and its non-normal cannot be linearized through mathematical transformations, we also do a further regression test by creating a three-category money variable defined as (a) no money, (b) money below the median, and (c) money above the median (Supplementary Tab. S11), which confirmed the simpler binary variable result which we report. A list and description of variables details please see Supplementary Tab. S12.

SI Figures

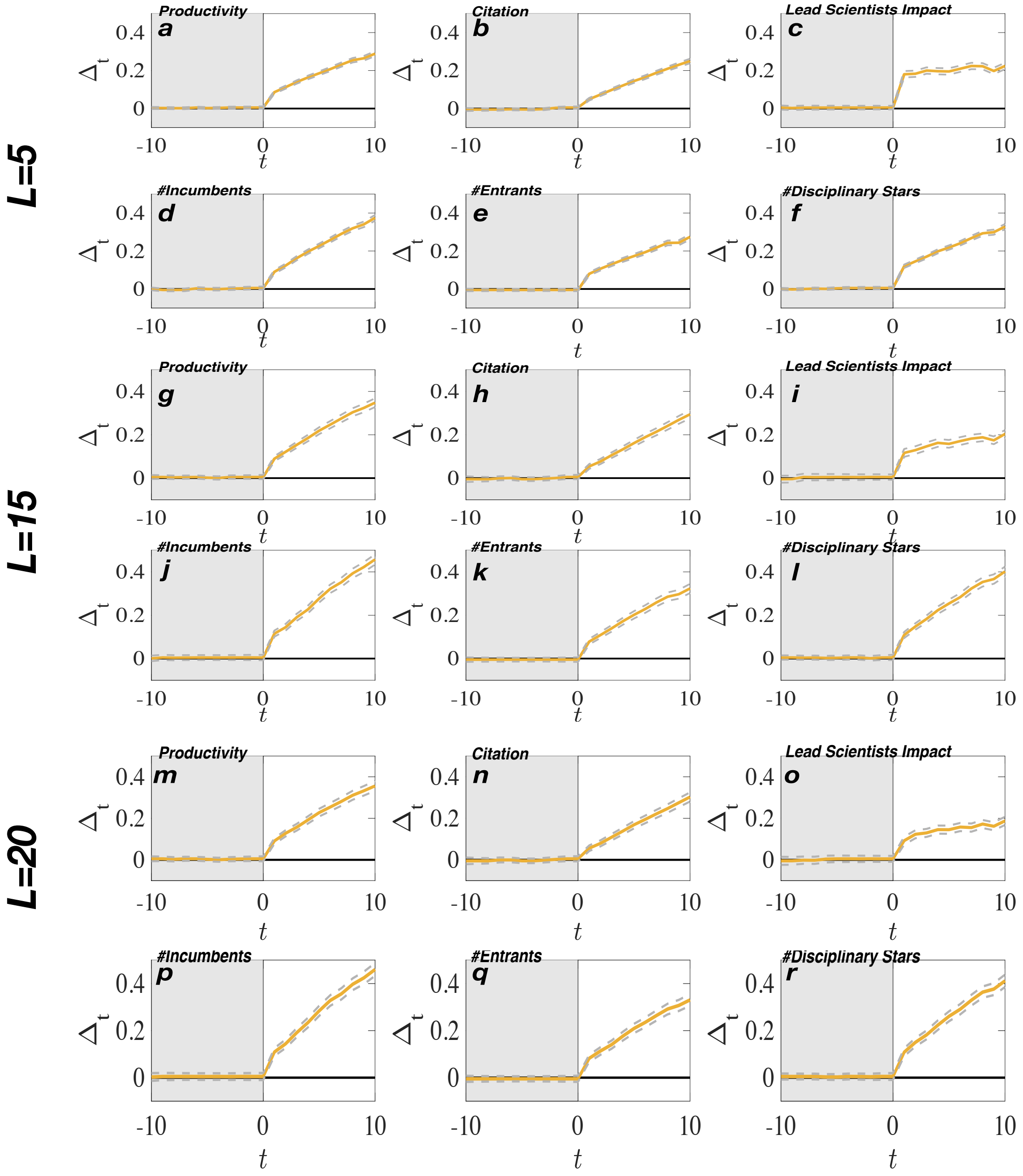

Supplementary Fig. S1. Main analysis with "known for" topics measured as >=5, >=15 or >=20 papers on a topic as opposed to >=10 papers. (a-r) Here we use an alternative criterion of the main topics for prizewinners by selecting **(a-f)** $L = 5$, **(g-l)** $L = 15$, and **(m-r)** $L = 20$. The findings are consistent with the main results. The dashed lines indicate the 95% confidence intervals.

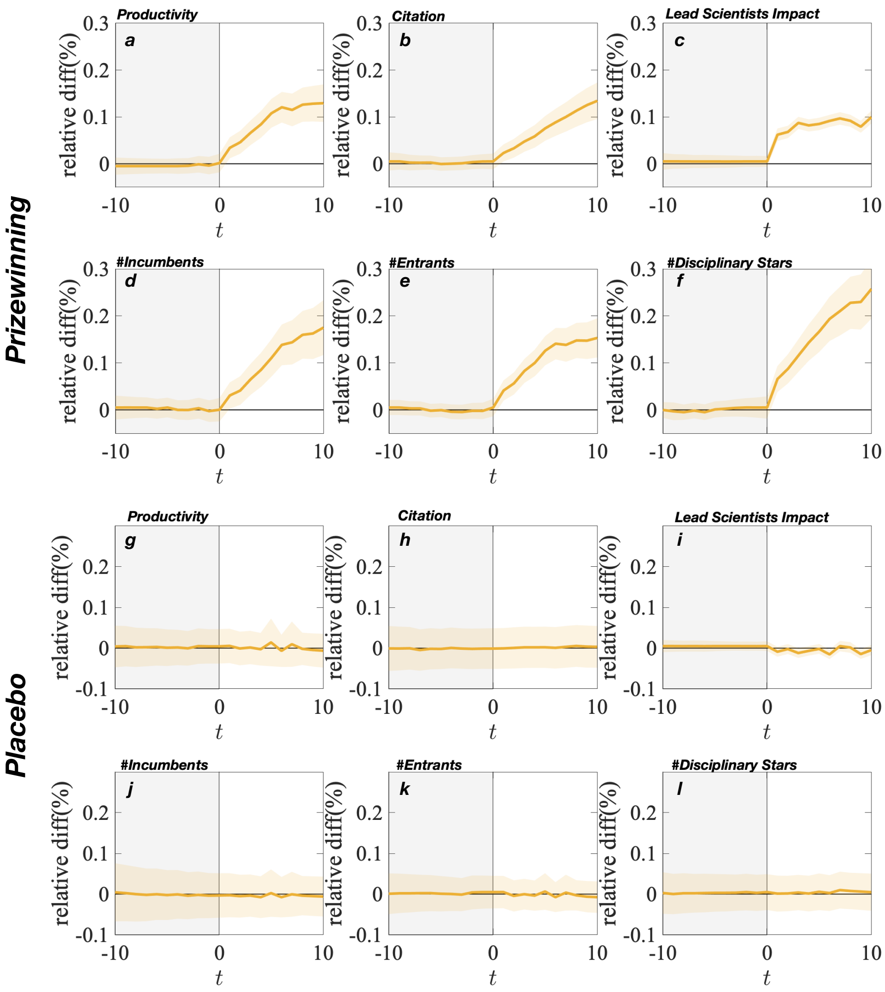

Supplementary Fig. S2. Alternative measures of extraordinary growth. (a-f) We repeat the main results with the alternative definition of difference. Here we calculate the ratio (relative) difference between the prizewinning topics and the expected growth, finding the main findings remain the same. **(g-l)** Placebo test for the analysis for results in a-e, where the growth pattern of the peer topic is flat before and after the prize. Error band indicates the 95% CI.

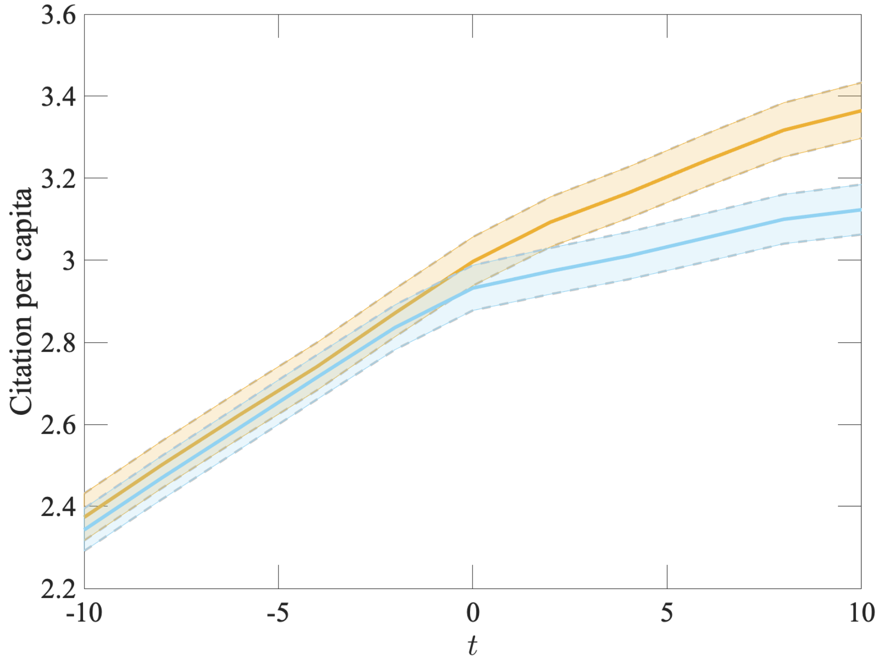

Supplementary Fig. S3. Citation per capita. Citations per capita increase after the prize, indicating the prize is also an important signal for research quality. Yellow curve corresponds to the prizewinning topics, and the blue curve represents the matched topics. Error band measures 95% CI.

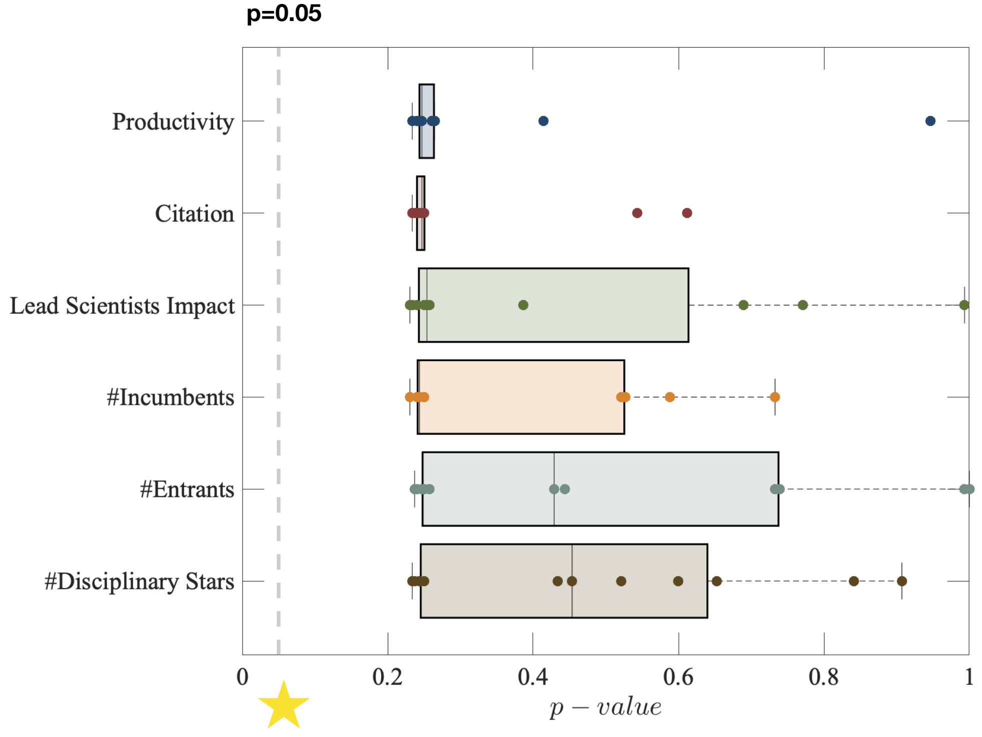

Supplementary Fig. S4. Prizewinning and Matched Non-prizewinning Peer Topics Have Statistically Indistinguishable Historical Growth Patterns Prior to the Prize Year. Based on six measures of a topic's growth (productivity, citation, lead scientist impact, #incumbents, #entrants and #disciplinary stars), we observed that for 11 consecutive years before the year the prizewinning topic is awarded its prize, the prizewinning topic and its peer topic have statistically indistinguishable growth patterns. Box plots show median and 90th and 10th percentiles of the p-value of the 11 two-tailed binomial tests, one for each year prior to the prize year. N=57,695 (11,539*5) topic pairs are observed for individual test. All tests of all measures (11*6==66 measures, dots) have a p-value > 0.05 (dashed line). The center line of the box plot is the median of the normalized grants, box limits correspond to the data's first and third quartiles, notches represent 95% CI,

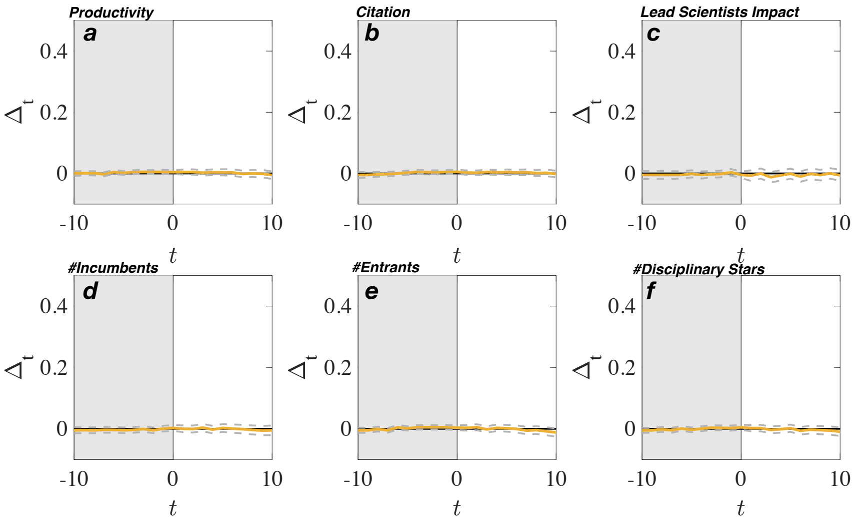

Supplementary Fig. S5. Placebo Tests. For each prizewinning topic, we select a non-prizewinning topic from the matching candidate pool as a "fake prizewinning topic". We repeat the DOM matching process for this faked topic, and search for the five peers for it. We perform the same analysis as we did for the prizewinning topic, finding that for these faked topics, there is no significant difference before and after the prizewinning event, validating our DOM method.

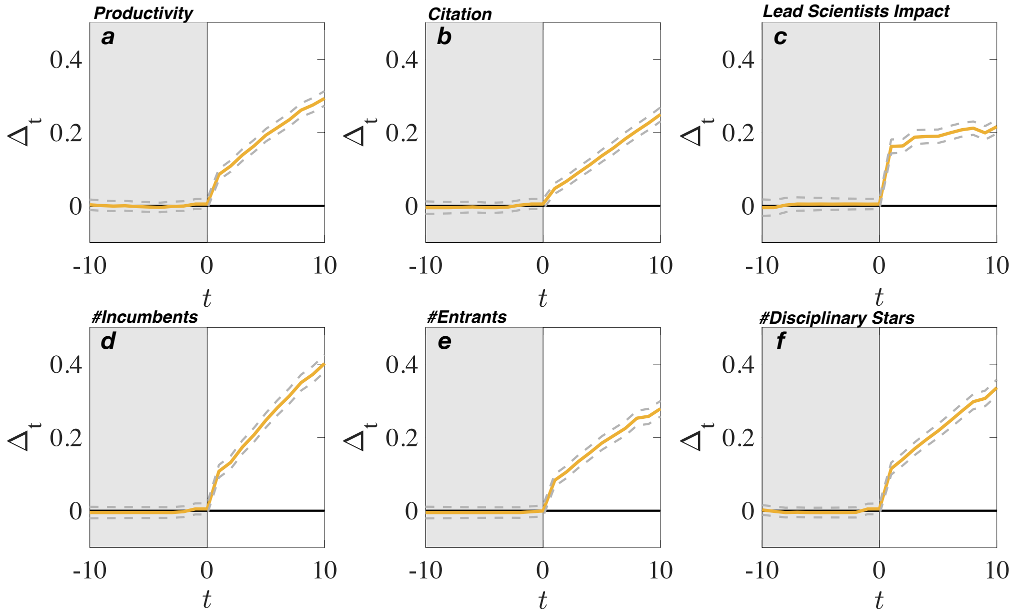

Supplementary Fig. S6. Using Mahalanobis distance in the matching procedure. (a-f) We repeat the main results with the alternative measure (Mahalanobis distance) to quantify the closeness of topics. We rematch each prizewinning topic with this new distance measure, repeating the main analysis, finding consistent growth patterns.

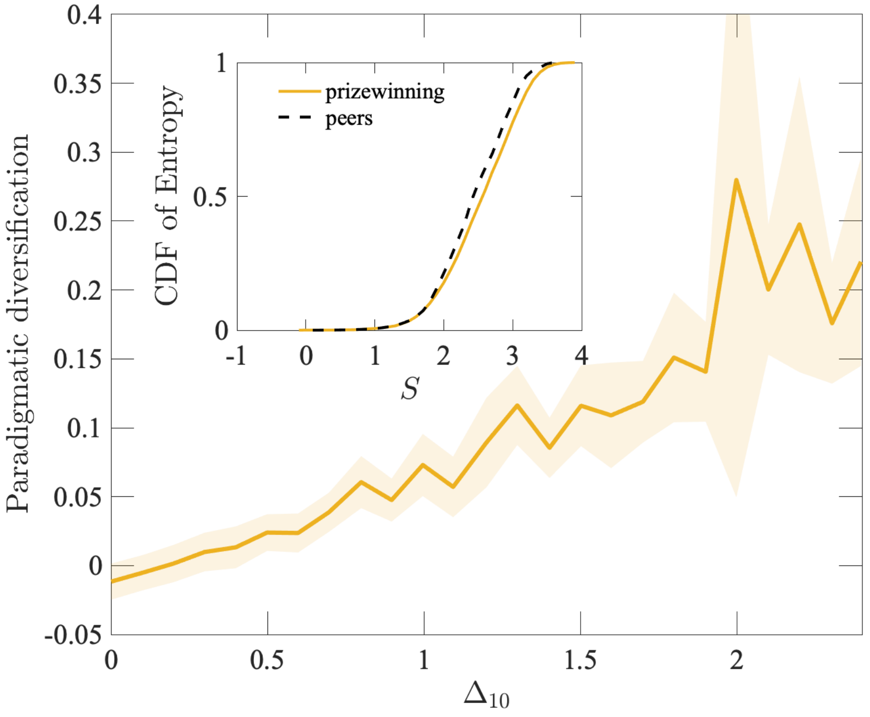

Supplementary Fig. S7. Prizes and Paradigmatic Diversification. The plot shows the percentage increase in paradigmatic diversification for prizewinning topics relative to peer topics. The inset shows the cumulative distribution of the paradigmatic diversity of the prizewinning topics and their peer groups significantly differ (K-S tst, two tailed test, $p = 3.1 * 10^{-25}$). The relationship between extraordinary growth and paradigmatic diversity shows that as Δ_{10} increases, relative paradigmatic diversity increases significantly (slope=0.109, OLS Regression, $p = 8.7 * 10^{-218}$). For example, when Δ_{10} equal to 1.5, a prizewinning topic is estimated to be 11.6% more diverse paradigmatical than its peer topics. Error band corresponds to 95% CI.

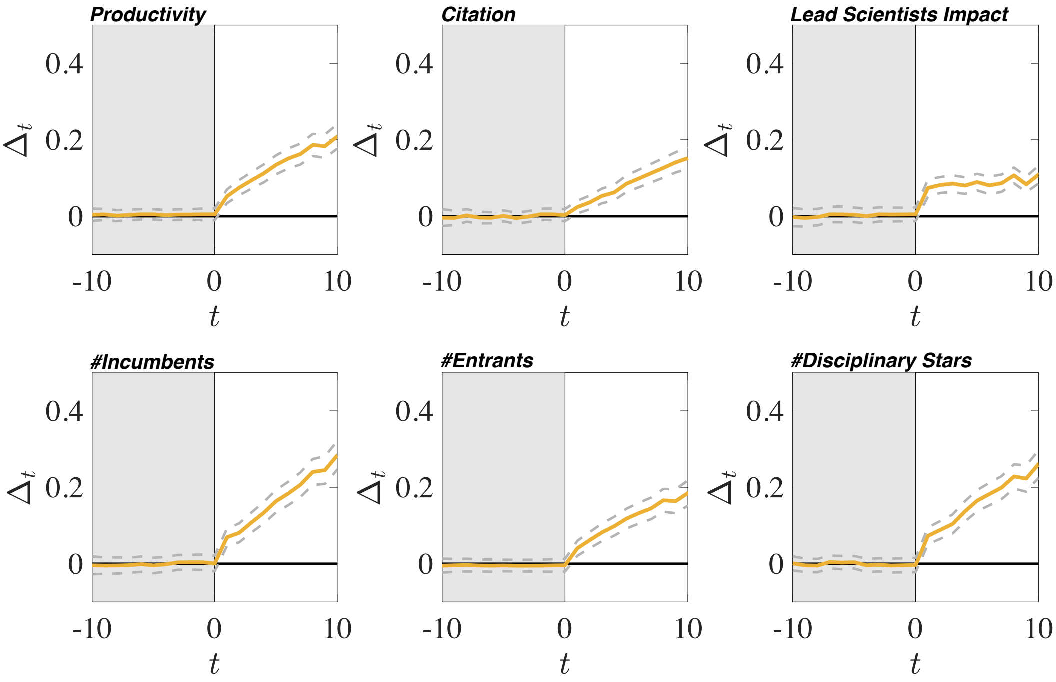

Supplementary Fig. S8. NIH subsample analysis (both prizewinning and peer topics are NIH funded). This figure reports a new analysis where both the prizewinning and peer topics are NIH funded topics. Of the 2,853 NIH funded prizewinning topics, 2,569 prizewinning topics were matched with five non-prizewinning, NIH funded peer topics using DOM. This procedure ensures that 100% of the peer topics are also NIH funded topics and the subsample is balanced and meets the parallel trend requirement. By repeating this matching process in this NIH funded topic pool, we find prizewinning topics grow relatively larger than peer topics after the prize year, in consistent with the main results reported in the main manuscript.

	Differences Between Prizewinning and Peer Topics' Big Five Growth Measures for 10 Years Before and After Prize	
	10 years Before the Prize	10 years After the Prize
**Annual Growth Measure**	**Binomial test**	**Binomial test**
1. productivity	0.5024 (ns)	0.6159***
2. citation	0.5025 (ns)	0.5976***
3. lead scientist impact	0.5008 (ns)	0.5821***
4. #incumbents	0.5024 (ns)	0.6238***
5. #entrants	0.5007 (ns)	0.5987***
6. #disciplinary stars	0.5025 (ns)	0.6177***
All tests 2-tailed. Binomial tests *** p<0.001		

Supplementary Table S1. Binomial tests for extraordinary growths after prizewinning events. For each of our 11,539 prizewinning topics and their peer topics, we calculate the fraction of pairs where the prizewinning topic grows faster than the peer topic, we find around 60% of the prizewinning topics are likelier to growth faster than their specific peer topics (binomial test, all *p* values<0.001), indicating that the link between prizewinning and extraordinary growth generalizes on the topic-by-topic level. As a comparison, the same analysis has been performed for the data 10 years before the prize, we find the fraction of topics with a positive growth is no significant different with 0.5 (two-tailed binomial test, all p values> 0.2, specific p-value see Fig. S4), indicating the difference between the prizewinning topics and the peer topics are well balanced before the prizewinning year, further validating our DOM method.

	Productivity	Citations	Impact of Topic's Lead Scientists	#Incumbents	#Entrants	#Disciplinay Stars working on the topic
**Recency**	0.0139***	0.0134***	0.00905***	0.0167***	0.0141***	0.0146***
	(0.000978)	(0.000955)	(0.000936)	(0.00116)	(0.00103)	(0.00109)
**Money (Yes/No)**	0.0393**	0.0329*	-0.0210	0.0413*	0.0391*	0.0401*
	(0.0146)	(0.0131)	(0.0162)	(0.0177)	(0.0157)	(0.0166)
**Discipline/General**	0.0705***	0.103***	0.0520**	0.0869***	0.0876***	0.127***
	(0.0165)	(0.0151)	(0.0195)	(0.0202)	(0.0177)	(0.0189)
**Prizewinner top**	-0.0239	-0.0151	-0.0126	-0.0189	-0.0283	-0.0234
	(0.0158)	(0.0141)	(0.0167)	(0.0192)	(0.0168)	(0.0176)
**Prize Age**	0.0000716	0.0000989	0.00000621	0.000182	0.0000124	0.0000498
	(0.0000830)	(0.0000782)	(0.000122)	(0.0000935)	(0.0000933)	(0.000114)
**Conferral times**	0.000286	0.000672***	0.000140	0.000260	0.000275	0.000331
	(0.000207)	(0.000181)	(0.000203)	(0.000245)	(0.000221)	(0.000235)
**Pageviews**	0.00000251	0.00000176	-0.000000124	0.00000333	0.00000290*	0.00000252
	(0.00000138)	(0.00000119)	(0.00000159)	(0.00000180)	(0.00000147)	(0.00000167)
**Discipline**	Yes	Yes	Yes	Yes	Yes	Yes
**Prizewinning year**	Yes	Yes	Yes	Yes	Yes	Yes
**Multiple recipients**	Yes	Yes	Yes	Yes	Yes	Yes
**Lagged variable**	Yes	Yes	Yes	Yes	Yes	Yes
**const**	0.662***	0.482***	0.542***	0.827***	0.703***	0.700***
	(0.0880)	(0.0774)	(0.123)	(0.101)	(0.0991)	(0.101)
**R-square**	0.318	0.374	0.120	0.267	0.295	0.299

* p<0.05, ** p<0.01, *** p<0.001

Supplementary Table S2: Regression analysis with robust standard errors of the relations between Prize Characteristics and Magnitude of Extraordinary Growth. OLS regression are used in these analyses.

	Productivity	Citations	Impact of Topic's Lead Scientists	#Incumbents	#Entrants	#Disciplinay Stars working on the topic
**Recency**	0.143***	0.146***	0.092***	0.146***	0.138***	0.134***
	(0.000978)	(0.000955)	(0.000936)	(0.00116)	(0.00103)	(0.00109)
**Money (Yes/No)**	0.021**	0.019*	-0.011	0.019*	0.020*	0.020*
	(0.0146)	(0.0131)	(0.0162)	(0.0177)	(0.0157)	(0.0166)
**Discipline/General**	0.034***	0.053***	0.025**	0.036***	0.040***	0.055***
	(0.0165)	(0.0151)	(0.0195)	(0.0202)	(0.0177)	(0.0189)
**Prizewinner top**	-0.012	-0.008	-0.006	-0.008	-0.013	-0.010
	(0.0158)	(0.0141)	(0.0167)	(0.0192)	(0.0168)	(0.0176)
**Prize Age**	0.005	0.008	0.000	0.011	0.001	0.003
	(0.0000830)	(0.0000782)	(0.000122)	(0.0000935)	(0.0000933)	(0.000114)
**Conferral times**	0.011	0.028***	0.005	0.009	0.010	0.012
	(0.000207)	(0.000181)	(0.000203)	(0.000245)	(0.000221)	(0.000235)
**Pageviews**	0.015	0.011	-0.001	0.017	0.016*	0.013
	(0.00000138)	(0.00000119)	(0.00000159)	(0.00000180)	(0.00000147)	(0.00000167)
**Discipline**	Yes	Yes	Yes	Yes	Yes	Yes
**Prizewinning year**	Yes	Yes	Yes	Yes	Yes	Yes
**Multiple recipients**	Yes	Yes	Yes	Yes	Yes	Yes
**Lagged variable**	Yes	Yes	Yes	Yes	Yes	Yes
**R-square**	0.318	0.374	0.120	0.267	0.295	0.299

* $p<0.05$, ** $p<0.01$, *** $p<0.001$

Supplementary Table S3: Regression analysis with robust standard errors of the relations between Prize Characteristics and Magnitude of Extraordinary Growth (Beta Coefficients). OLS regression are used in these analyses.

	Productivity	Productivity	Productivity	Productivity	Productivity
**Recency**	0.0140***				0.0139***
	(0.000977)				(0.000978)
**Money (Yes/No)**		0.0554***		0.0565***	0.0393**
		(0.0146)		(0.0146)	(0.0146)
**Discipline/General**			0.0640***	0.0654***	0.0705***
			(0.0167)	(0.0167)	(0.0165)
**Prizewinner top**	-0.0213	-0.00344	-0.00541	-0.00577	-0.0239
	(0.0158)	(0.0159)	(0.0160)	(0.0159)	(0.0158)
**Prize Age**	0.0000181	0.0000742	0.0000522	0.0000964	0.0000716
	(0.0000824)	(0.0000797)	(0.0000796)	(0.0000798)	(0.0000830)
**Conferral times**	0.0000797	-0.0000550	0.000178	0.000154	0.000286
	(0.000198)	(0.000198)	(0.000207)	(0.000207)	(0.000207)
**Pageviews**	0.00000229	0.00000211	0.00000330*	0.00000269	0.00000251
	(0.00000138)	(0.00000141)	(0.00000140)	(0.00000141)	(0.00000138)
**Discipline**	Yes	Yes	Yes	Yes	Yes
**Prizewinning year**	Yes	Yes	Yes	Yes	Yes
**Multiple recipients**	Yes	Yes	Yes	Yes	Yes
**Lagged variable**	Yes	Yes	Yes	Yes	Yes
**const**	0.752***	0.601***	0.546***	0.529***	0.662***
	(0.0848)	(0.0859)	(0.0887)	(0.0889)	(0.0880)
**R-square**	0.316	0.299	0.299	0.300	0.318
ΔBIC	-312.74	-5.05	-4.33	-9.97	-318.04

* p<0.05, ** p<0.01, *** p<0.001

Supplementary Table S4: Regression analysis with robust standard errors of the relations between Prize Characteristics and Magnitude of Extraordinary Growth (Productivity). OLS regression are used in these analyses.

	Citations	Citations	Citations	Citations	Citations
**Recency**	0.0134***				0.0134***
	(0.000956)				(0.000955)
**Money (Yes/No)**		0.0478***		0.0495***	0.0329*
		(0.0131)		(0.0131)	(0.0131)
**Discipline/General**			0.0966***	0.0978***	0.103***
			(0.0152)	(0.0152)	(0.0151)
**Prizewinner top**	-0.0114	0.00583	0.00266	0.00235	-0.0151
	(0.0142)	(0.0143)	(0.0143)	(0.0143)	(0.0141)
**Prize Age**	0.0000398	0.0000896	0.0000840	0.000123	0.0000989
	(0.0000775)	(0.0000766)	(0.0000767)	(0.0000767)	(0.0000782)
**Conferral times**	0.000358*	0.000231	0.000565**	0.000545**	0.000672***
	(0.000174)	(0.000174)	(0.000184)	(0.000183)	(0.000181)
**Pageviews**	0.00000118	0.00000107	0.00000247*	0.00000194	0.00000176
	(0.00000118)	(0.00000121)	(0.00000120)	(0.00000121)	(0.00000119)
**Discipline**	Yes	Yes	Yes	Yes	Yes
**Prizewinning year**	Yes	Yes	Yes	Yes	Yes
**Multiple recipients**	Yes	Yes	Yes	Yes	Yes
**Lagged variable**	Yes	Yes	Yes	Yes	Yes
**const**	0.0134***	0.0134***	0.0134***	0.0134***	0.0134***
	(0.000955)	(0.000955)	(0.000955)	(0.000955)	(0.000955)
**R-square**	0.372	0.353	0.355	0.355	0.374
ΔBIC	-355.67	-3.85	-29.10	-33.92	-387.55

* p<0.05, ** p<0.01, *** p<0.001

Supplementary Table S5: Regression analysis with robust standard errors of the relations between Prize Characteristics and Magnitude of Extraordinary Growth (Citations). OLS regression are used in these analyses.

	Impact of Topic's Lead Scientists	Impact of Topic's Lead Scientists	Impact of Topic's Lead Scientists	Impact of Topic's Lead Scientists	Impact of Topic's Lead Scientists
**Recency**	0.00894***				0.00905***
	(0.000927)				(0.000936)
**Money (Yes/No)**		-0.0106		-0.00977	-0.0210
		(0.0161)		(0.0161)	(0.0162)
**Discipline/General**			0.0488*	0.0486*	0.0520**
			(0.0196)	(0.0196)	(0.0195)
**Prizewinner top**	-0.0108	0.000953	-0.000839	-0.000777	-0.0126
	(0.0166)	(0.0167)	(0.0168)	(0.0168)	(0.0167)
**Prize Age**	0.00000564	0.00000588	0.0000300	0.0000224	0.00000621
	(0.000122)	(0.000118)	(0.000118)	(0.000118)	(0.000122)
**Conferral times**	-0.0000380	-0.000102	0.0000498	0.0000538	0.000140
	(0.000192)	(0.000191)	(0.000202)	(0.000202)	(0.000203)
**Pageviews**	-0.000000823	-0.000000436	-0.000000106	-2.02e-09	-0.000000124
	(0.00000157)	(0.00000159)	(0.00000160)	(0.00000161)	(0.00000159)
**Discipline**	Yes	Yes	Yes	Yes	Yes
**Prizewinning year**	Yes	Yes	Yes	Yes	Yes
**Multiple recipients**	Yes	Yes	Yes	Yes	Yes
**Lagged variable**	Yes	Yes	Yes	Yes	Yes
**const**	0.592***	0.509***	0.452***	0.455***	0.542***
	(0.120)	(0.120)	(0.122)	(0.122)	(0.123)
**R-square**	0.120	0.112	0.113	0.113	0.120
ΔBIC	-91.7	9.00	3.32	12.29	-387.55

* p<0.05, ** p<0.01, *** p<0.001

Supplementary Table S6: Regression analysis with robust standard errors of the relations between Prize Characteristics and Magnitude of Extraordinary Growth (Impact of Topic's Lead Scientists). OLS regression are used in these analyses.

	#Incumbents	#Incumbents	#Incumbents	#Incumbents	#Incumbents
**Recency**	0.0167***				0.0167***
	(0.00116)				(0.00116)
**Money (Yes/No)**		0.0606***		0.0620***	0.0413*
		(0.0177)		(0.0177)	(0.0177)
**Discipline/General**			0.0793***	0.0808***	0.0869***
			(0.0205)	(0.0205)	(0.0202)
**Prizewinner top**	-0.0157	0.00584	0.00336	0.00297	-0.0189
	(0.0192)	(0.0193)	(0.0193)	(0.0193)	(0.0192)
**Prize Age**	0.000121	0.000184*	0.000163	0.000211*	0.000182
	(0.0000927)	(0.0000921)	(0.0000919)	(0.0000922)	(0.0000935)
**Conferral times**	0.00000193	-0.000157	0.000127	0.000102	0.000260
	(0.000234)	(0.000235)	(0.000246)	(0.000246)	(0.000245)
**Pageviews**	0.00000299	0.00000284	0.00000422*	0.00000356	0.00000333
	(0.00000179)	(0.00000181)	(0.00000181)	(0.00000182)	(0.00000180)
**Discipline**	Yes	Yes	Yes	Yes	Yes
**Prizewinning year**	Yes	Yes	Yes	Yes	Yes
**Multiple recipients**	Yes	Yes	Yes	Yes	Yes
**Lagged variable**	Yes	Yes	Yes	Yes	Yes
**const**	0.935***	0.756***	0.686***	0.667***	0.827***
	(0.0978)	(0.0991)	(0.102)	(0.102)	(0.101)
**R-square**	0.266	0.248	0.248	0.249	0.267
$\boldsymbol{\Delta BIC}$	-302.16	-2.22	-4.75	-7.50	-305.94

* p<0.05, ** p<0.01, *** p<0.001

Supplementary Table S7: Regression analysis with robust standard errors of the relations between Prize Characteristics and Magnitude of Extraordinary Growth (#Incumbents). OLS regression are used in these analyses.

	#Entrants	#Entrants	#Entrants	#Entrants	#Entrants
**Recency**	0.0142***				0.0141***
	(0.00103)				(0.00103)
**Money (Yes/No)**		0.0552***		0.0566***	0.0391*
		(0.0157)		(0.0157)	(0.0157)
**Discipline/General**			0.0810***	0.0824***	0.0876***
			(0.0179)	(0.0179)	(0.0177)
**Prizewinner top**	-0.0251	-0.00691	-0.00949	-0.00984	-0.0283
	(0.0168)	(0.0169)	(0.0169)	(0.0169)	(0.0168)
**Prize Age**	-0.0000465	0.00000967	-0.00000665	0.0000376	0.0000124
	(0.0000927)	(0.0000913)	(0.0000907)	(0.0000911)	(0.0000933)
**Conferral times**	0.0000135	-0.000123	0.000164	0.000141	0.000275
	(0.000211)	(0.000210)	(0.000221)	(0.000220)	(0.000221)
**Pageviews**	0.00000253	0.00000236	0.00000370*	0.00000309*	0.00000290*
	(0.00000146)	(0.00000149)	(0.00000148)	(0.00000149)	(0.00000147)
**Discipline**	Yes	Yes	Yes	Yes	Yes
**Prizewinning year**	Yes	Yes	Yes	Yes	Yes
**Multiple recipients**	Yes	Yes	Yes	Yes	Yes
**Lagged variable**	Yes	Yes	Yes	Yes	Yes
**const**	0.811***	0.659***	0.585***	0.568***	0.703***
	(0.0957)	(0.0965)	(0.0992)	(0.0996)	(0.0991)
**R-square**	0.294	0.278	0.278	0.279	0.295
ΔBIC	-279.28	-3.10	-9.76	-13.51	-289.35

* p<0.05, ** p<0.01, *** p<0.001

Supplementary Table S8: Regression analysis with robust standard errors of the relations between Prize Characteristics and Magnitude of Extraordinary Growth (#Entrants). OLS regression are used in these analyses.

	#Disciplinay Stars working on the topic	#Disciplinay Stars working on the topic	#Disciplinay Stars working on the topic	#Disciplinay Stars working on the topic	#Disciplinay Stars working on the topic
**Recency**	0.0146***				0.0146***
	(0.00109)				(0.00109)
**Money (Yes/No)**		0.0562***		0.0582***	0.0401*
		(0.0166)		(0.0166)	(0.0166)
**Discipline/General**			0.120***	0.121***	0.127***
			(0.0192)	(0.0192)	(0.0189)
**Prizewinner top**	-0.0188	-0.0000501	-0.00399	-0.00436	-0.0234
	(0.0176)	(0.0177)	(0.0177)	(0.0177)	(0.0176)
**Prize Age**	-0.0000226	0.0000347	0.0000303	0.0000758	0.0000498
	(0.000114)	(0.000110)	(0.000108)	(0.000109)	(0.000114)
**Conferral times**	-0.0000554	-0.000195	0.000217	0.000193	0.000331
	(0.000225)	(0.000225)	(0.000235)	(0.000235)	(0.000235)
**Pageviews**	0.00000180	0.00000164	0.00000334*	0.00000272	0.00000252
	(0.00000165)	(0.00000169)	(0.00000168)	(0.00000169)	(0.00000167)
**Discipline**	Yes	Yes	Yes	Yes	Yes
**Prizewinning year**	Yes	Yes	Yes	Yes	Yes
**Multiple recipients**	Yes	Yes	Yes	Yes	Yes
**Lagged variable**	Yes	Yes	Yes	Yes	Yes
**const**	0.851***	0.694***	0.578***	0.560***	0.700***
	(0.0986)	(0.0992)	(0.101)	(0.102)	(0.101)
**R-square**	0.297	0.281	0.283	0.283	0.299
ΔBIC	-266.40	-2.24	-28.30	-31.44	-296.11

* p<0.05, ** p<0.01, *** p<0.001

Supplementary Table S9: Regression analysis with robust standard errors of the relations between Prize Characteristics and Magnitude of Extraordinary Growth (#Disciplinay Stars working on the topic). OLS regression are used in these analyses.

	Productivity	Citations	Impact of Topic's Lead Scientists	#Incumbents	#Entrants	#DisciplinayStars working on the topic
Est1 r2	0.321	0.352	0.111	0.263	0.262	0.296
Est2 r2	0.278	0.363	0.092	0.283	0.277	0.294
Est3 r2	0.309	0.359	0.105	0.232	0.265	0.317
Est4 r2	0.354	0.383	0.107	0.228	0.302	0.260
Est5 r2	0.304	0.383	0.117	0.242	0.322	0.251
Est6 r2	0.316	0.337	0.103	0.296	0.331	0.281
Est7 r2	0.307	0.381	0.106	0.292	0.277	0.319
Est8 r2	0.323	0.336	0.089	0.198	0.307	0.274
Est9 r2	0.273	0.384	0.130	0.282	0.258	0.326
Est10 r2	0.291	0.382	0.094	0.251	0.251	0.282
All data r2	0.318	0.375	0.120	0.267	0.296	0.300

Supplementary Table S10. 10-Fold Cross-Validation for Regression Results.

	Productivity	Citations	Impact of Topic's Lead Scientists	#Incumbents	#Entrants	#Disciplinay Stars working on the topic
**No money**	0	0	0	0	0	0
	(.)	(.)	(.)	(.)	(.)	(.)
**Low money**	0.0451*	0.0122	-0.0419	0.0703**	0.0157	0.0301
	(0.0214)	(0.0199)	(0.0216)	(0.0252)	(0.0223)	(0.0242)
**High Money**	0.0517**	0.0754***	0.00419	0.0363	0.0718***	0.0578**
	(0.0196)	(0.0185)	(0.0197)	(0.0230)	(0.0209)	(0.0218)
**Const**	0.328***	0.277***	0.231***	0.433***	0.305***	0.383***
	(0.0117)	(0.0110)	(0.0121)	(0.0139)	(0.0124)	(0.0130)

* p<0.05, ** p<0.01, *** p<0.001

Supplementary Tab S11. Robustness check of the relations between Prize Characteristics and the money of prizes. We identified the median of money amounts for prizes with money and we separate prizes into three groups. OLS regression are used in these analyses.

	Description
**Recency**	How many years the prizewinner has been working on the topic before the prizewinning year. (mean=11.40, STD=9.46)
**Money**	This dummy variable takes on the value of 1 if prize money is associated with the prize and 0 otherwise (45% of the prizes contains money).
**Discipline-specific or General prize**	This dummy variable takes on the value of 1 if at least 85% of all the winners of the prize come from the same discipline and 0 otherwise (78% are discipline-specific prizes).
**Prizewinner top**	This dummy variable takes on the value of 1 if the prizewinner is among top 5% scientists within the topic in the prizewinning year based on citation records and 0 otherwise. (28.3% of the prizewinners are among the top 5%).
**Prize Age**	The age of the prize in years before the prizewinning year (mean=27.47, STD=29.94).
**Number of times the prize was conferred**	The number of times the prize was bestowed before the prizewinning year (mean=26.08, STD=36.14).
**Pageviews**	The number of wikipedia pageviews of the prize before the end of 2017.
**Discipline**	This categorical variable is used to define each of 19 separate disciplines.
**Prizewinning year**	This categorical variable is used to define the calendar year of the prizewinning event (1970-2007).
**Multiple recipients**	This dummy variable takes on the value of 1 if there are multiple prizewinners for the topic in the prizewinning year and 0 otherwise (4.8% of the topics have multiple prizewinners).
**Lagged dependent variables**	Lagged values of each growth trend at times t-1, t-2, and t-3 years.

Supplementary Tab S12. Description of Regression Variables and Their Statistics.

A Topic's Comparative Post-Prizewinning Growth on Six Measures – Both prizewinning topics and peer topics are NIH funded

	(1)	(2)	(3)	(4)	(5)	(6)
	**Productivity**	**Citations**	**Impact of Topic's Lead Scientists**	**#Incumbents**	**#Entrants**	**#Disciplinay Stars working on the topic**
Prizewinning (β_1)	0.00406	-0.000521	0.00212	-0.00111	-0.00453	-0.00189
	(0.0590)	(0.0598)	(0.0257)	(0.0678)	(0.0624)	(0.0540)
Post (β_2)	0.641***	1.384***	0.960***	1.048***	0.787***	0.796***
	(0.0189)	(0.0224)	(0.0132)	(0.0240)	(0.0191)	(0.0204)
Prizewinning * Post (β_3)	0.132***	0.0896***	0.0856***	0.173***	0.124***	0.166***
	(0.0230)	(0.0269)	(0.0175)	(0.0290)	(0.0235)	(0.0246)
Fixed Effect Controls:						
Discipline	Yes	Yes	Yes	Yes	Yes	Yes
Prizewinning Year	Yes	Yes	Yes	Yes	Yes	Yes
const	3.806***<br>(0.150)	6.299***<br>(0.146)	7.098***<br>(0.0582)	2.792***<br>(0.167)	4.415***<br>(0.146)	2.397***<br>(0.128)
N	323,694	323,694	323,694	323,694	323,694	323,694
R-sq	0.213	0.318	0.327	0.251	0.227	0.268

Standard errors in parentheses. * p<0.05, ** p<0.01, *** p<0.001

Supplementary Tab S13: DID analysis of a prizewinning topic's comparative post-prizewinning growth on six measures for topics that received NIH funding (both prizewinning and peer topics). This table reported a new analysis where both the prizewinning and peer topics are NIH funded topics. Of the 2,853 NIH funded prizewinning topics, 2,569 prizewinning topics were matched with five non-prizewinning, NIH funded peer topics using DOM. This procedure ensures that 100% of the peer topics are also NIH funded topics and the subsample is balanced and meets the parallel trend requirement. By repeating the matching process in this NIH funded topic pool, we find the results are consistent**. OLS regression are used in these analyses.**

References:

1 Ma, Y. & Uzzi, B. Scientific prize network predicts who pushes the boundaries of science. *Proceedings of the National Academy of Sciences* **115**, 12608-12615 (2018).

2 Li, J., Yin, Y., Fortunato, S. & Wang, D. A dataset of publication records for Nobel laureates. *Sci Data* **6**, 33, doi:10.1038/s41597-019-0033-6 (2019).

3 Frank, M. R. *et al.* Toward understanding the impact of artificial intelligence on labor. *Proc Natl Acad Sci U S A* **116**, 6531-6539, doi:10.1073/pnas.1900949116 (2019).

4 Goh, K.-I. *et al.* The human disease network. *Proceedings of the National Academy of Sciences* **104**, 8685-8690 (2007).

5 Sinatra, R., Wang, D., Deville, P., Song, C. & Barabási, A.-L. J. S. Quantifying the evolution of individual scientific impact. **354**, aaf5239 (2016).

6 Liu, L. *et al.* Hot streaks in artistic, cultural, and scientific careers. *Nature* **559**, 396 (2018).

7 Jin, G. Z., Jones, B., Lu, S. F. & Uzzi, B. The reverse Matthew effect: Consequences of retraction in scientific teams. *Review of Economics and Statistics* **101**, 492-506 (2019).

8 Lu, S. F., Zhe Jin, G., Uzzi, B. & Jones, B. The Retraction Penalty: Evidence from the Web of Science. *Nature Scientific Reports* **3** (2013).

9 Pimentel, S. D., Kelz, R. R., Silber, J. H. & Rosenbaum, P. R. Large, sparse optimal matching with refined covariate balance in an observational study of the health outcomes produced by new surgeons. *Journal of the American Statistical Association* **110**, 515-527 (2015).

10 Rosenbaum, P. R. Optimal matching for observational studies. *Journal of the American Statistical Association* **84**, 1024-1032 (1989).

11 Rosenbaum, P. R. Modern Algorithms for Matching in Observational Studies. *Annual Review of Statistics and Its Application* **7** (2019).

12 Stuart, E. A. Matching methods for causal inference: A review and a look forward. *Statistical science: a review journal of the Institute of Mathematical Statistics* **25**, 1 (2010).

13 Zubizarreta, J. R. Using mixed integer programming for matching in an observational study of kidney failure after surgery. *Journal of the American Statistical Association* **107**, 1360-1371 (2012).

14 Kuhn, T. S. *The Structure of Scientific Revolutions*. (University of Chicago, 1970).

15 Jones, B. F. The burden of knowledge and the 'Death of the Renaissance Man': Is innovation getting harder? *Review of Economic Studies* (2008).
