# Supplementary material for: Scientific prizes and the extraordinary growth of scientific topics
Source: Nat Commun. 2021 Oct 5;12:5619. doi: 10.1038/s41467-021-25712-2 (PMC8492701; doi:10.1038/s41467-021-25712-2)
Supplement: Supplementary file 2 — Reporting Summary [file 41467_2021_25712_MOESM2_ESM.pdf]

nature research

Corresponding author(s): Brian Uzzi

Last updated by author(s): 07/31/2021

Reporting Summary

Nature Research wishes to improve the reproducibility of the work that we publish. This form provides structure for consistency and transparency in reporting. For further information on Nature Research policies, see our Editorial Policies and the Editorial Policy Checklist.

Statistics

For all statistical analyses, confirm that the following items are present in the figure legend, table legend, main text, or Methods section.

n/a	Confirmed	
☐	☒	The exact sample size (n) for each experimental group/condition, given as a discrete number and unit of measurement
☐	☒	A statement on whether measurements were taken from distinct samples or whether the same sample was measured repeatedly
☐	☒	The statistical test(s) used AND whether they are one- or two-sided<br>*Only common tests should be described solely by name; describe more complex techniques in the Methods section.*
☐	☒	A description of all covariates tested
☐	☒	A description of any assumptions or corrections, such as tests of normality and adjustment for multiple comparisons
☐	☒	A full description of the statistical parameters including central tendency (e.g. means) or other basic estimates (e.g. regression coefficient) AND variation (e.g. standard deviation) or associated estimates of uncertainty (e.g. confidence intervals)
☐	☒	For null hypothesis testing, the test statistic (e.g. F, t, r) with confidence intervals, effect sizes, degrees of freedom and P value noted<br>*Give P values as exact values whenever suitable.*
☒	☐	For Bayesian analysis, information on the choice of priors and Markov chain Monte Carlo settings
☒	☐	For hierarchical and complex designs, identification of the appropriate level for tests and full reporting of outcomes
☒	☐	Estimates of effect sizes (e.g. Cohen's d, Pearson's r), indicating how they were calculated

Our web collection on statistics for biologists contains articles on many of the points above.

Software and code

Policy information about availability of computer code

Data collection	Microsoft Academic Graph dataset was collected from Microsoft. Prizewinner dataset was collected from Wikipedia website. Data collection was conducted using Python 2.7.
Data analysis	Matlab R2017b, Matlab R2021a and Stata 16.0 are used to analyze the data. No custom software is used in our analysis.

For manuscripts utilizing custom algorithms or software that are central to the research but not yet described in published literature, software must be made available to editors and reviewers. We strongly encourage code deposition in a community repository (e.g. GitHub). See the Nature Research guidelines for submitting code & software for further information.

Data

Policy information about availability of data

All manuscripts must include a data availability statement. This statement should provide the following information, where applicable:

- Accession codes, unique identifiers, or web links for publicly available datasets
- A list of figures that have associated raw data
- A description of any restrictions on data availability

Data used in this work are publicly available from Wikipedia and Microsoft Academic Graph (MAG). The MAG dataset could be obtained from: https://www.microsoft.com/en-us/research/project/microsoft-academic-graph/. The Wikipedia information could be obtained from: https://en.wikipedia.org/wiki/Main_Page.

Field-specific reporting

Please select the one below that is the best fit for your research. If you are not sure, read the appropriate sections before making your selection.

- [ ] Life sciences
- [x] Behavioural & social sciences
- [ ] Ecological, evolutionary & environmental sciences

For a reference copy of the document with all sections, see nature.com/documents/nr-reporting-summary-flat.pdf

Behavioural & social sciences study design

All studies must disclose on these points even when the disclosure is negative.

Study description	Examine the statistical relationship between prizewinning and the extraordinary growth of scientific topics
Research sample	Data were collected on 405 scientific prizes conferred 2,900 times between 1970 and 2007 with respect to 11,539 scientific topics in 19 disciplines from Wikipedia pages, prize homepages, and Microsoft Academic Graph. This sample was chosen because of its size, diversity in terms of disciplinary breath, types, age, prestige, national origin, and use in prior research on prizes. Representativeness of the sample can only be determined by the degree to which it is a random sample, which is indeterminate. However, since anyone is free to create or edit a wikipedia page for a prize, the costs of creating a wikipedia page are low and without technical barriers to entry, and the sample is large and international it would appear that most recognized prizes would have a wikipage.
Sampling strategy	All relevant data from Wikipedia and MAG are collected without a further sampling process. The sample size in this paper was chosen to have large enough statistics for studying relationship between prizes and topics and follow closely the same procedure used by similar studies in the domain.
Data collection	Microsoft Academic Graph dataset was downloaded from Microsoft API. Prizewinner dataset was algorithmically collected from wikipedia website and manually checked by the researchers. This an exploratory study; no hypotheses were prespecified.
Timing	We did data collection of the wikipedia prizewinning datasets in Dec. 2016. The MAG dataset was downloaded in Nov. 2017. To avoid possible upgrade of the wikipedia data in the meantime, we did wikipedia prizewinning dataset collection again in Apr. 2018.
Data exclusions	The analysis has no data exclusion.
Non-participation	No dropout.
Randomization	No experiment in our study.

Reporting for specific materials, systems and methods

We require information from authors about some types of materials, experimental systems and methods used in many studies. Here, indicate whether each material, system or method listed is relevant to your study. If you are not sure if a list item applies to your research, read the appropriate section before selecting a response.

Materials & experimental systems

n/a	Involved in the study	
x		Antibodies
x		Eukaryotic cell lines
x		Palaeontology and archaeology
x		Animals and other organisms
x		Human research participants
x		Clinical data
x		Dual use research of concern

Methods

n/a	Involved in the study	
x		ChIP-seq
x		Flow cytometry
x		MRI-based neuroimaging
